# High Gastrointestinal Colonization Rate of Vancomycin-Resistant *Enterococci* among Hospitalized Patients: Potential Source for Resistant Gene

**DOI:** 10.1155/2024/6430026

**Published:** 2024-01-08

**Authors:** Techilo Habtemariam Mengesha, Musa Mohammed Ali, Mulugeta Mengistu, Demissie Assegu Fenta

**Affiliations:** ^1^Hawassa University, Comprehensive Specialized Hospital, Hawassa, Ethiopia; ^2^School of Medical Laboratory Science, College of Medicine and Health Sciences, Hawassa University, Hawassa, Ethiopia

## Abstract

**Background:**

Vancomycin-resistant *Enterococci* (VRE) is a global health problem and responsible for healthcare-associated infections (HAIs) in patients with prolonged hospital stay, severe underlying disease, and previous broad-spectrum antibiotic therapy. These bacteria can cross-resist and transfer drug-resistant genes to other potentially pathogenic bacteria. Therefore; this study was aimed to determine the gastrointestinal colonization rate of VRE, its antimicrobial susceptibility profile, and associated factors among hospitalized patients.

**Methods:**

Prospective cross-sectional study was conducted using stool samples from 223 patients admitted to different wards at Hawassa University Comprehensive Specialized Hospital, from April 1 to June 30, 2021. Patients admitted to the hospital for more than 48 hours for various medical conditions were included. Sociodemographic and clinical characteristics were collected using a structured questionnaire. Fecal specimens were cultured on *Enterococci* selective media. *Enterococcus* species were identified using their growth and mannitol fermentation properties. Vancomycin resistance was screened using both the Kirby–Bauer disk diffusion method and a vancomycin *E*-test strip. Data were entered and analyzed using SPSS version 25. Descriptive and logistic regressions were used to determine the frequency and association of factors with the VRE colonization rate. A *p* value of <0.05 was considered statistically significant.

**Results:**

A total of 223 fecal specimens were collected and processed, and 141 (63.2%) them were positive for *Enterococci*. The predominant species was *E. faecalis* 65 (46.1%) followed by *E. faecium* 76 (53.9%). In this study, the gastrointestinal colonization rate of VRE was 15 (6.7%) and all the species belong to *E*. *faecium*. Study participants who had no formal education (AOR = 4.26, 95% CI: 1.01, 18.06), hospitalized patients for >2 weeks (AOR = 4.10, 95% CI: 1.08, 15.57), and those who had a history of treatment with vancomycin (AOR = 4.77, 95% CI: 1.26, 18.09) were more likely to be colonized with vancomycin-resistant *Enterococci*. More than 95% of *Enterococci* isolates were susceptible to linezolid, whereas 70.2%, 63.1%, 56.7%, and 53.9% were resistant to tetracycline, erythromycin, penicillin, and ampicillin, respectively. Among the total *Enterococci* isolated, 141 (54.6%) were multidrug resistant.

**Conclusions:**

In our study, high proportion of vancomycin-resistant *Enterococci* was found. Previous exposure to antibiotics and hospital stay were significant factors for VRE gut colonization. The isolated *Enterococci* showed variable degrees of resistance to commonly prescribed antibiotics which leads to a worldwide problem multidrug resistance. Therefore, periodic surveillance on antimicrobial resistance pattern, adhering to rational use of antibiotics, and implementing infection prevention protocols may reduce colonization by VRE.

## 1. Introduction

*Enterococci* are natural inhabitants of the intestine, oral cavity, and the female genital tract of humans, and animals; however, they can cause opportunistic infections if they are relocated to sterile sites [1, 2]. In a hospital environment after gastrointestinal colonization, *Enterococci* can lead to various infections that include bloodstream infections, infective endocarditis, intra-abdominal/pelvic abscess, urinary tract, and surgical wound infection in critically ill patients [2]. Many of these infections originate from the intestinal flora of colonized individuals. VRE has different selection pressures for proliferation and rapid expansion of its resistant population [3].

*Enterococci* can grow under a wide range of temperatures and pH and are resistant to dry conditions. They can grow in a high salt concentration. As a result, *Enterococci* can persist in a hospital environment for a long period of time and spread easily among admitted patients and hospital staff [4].

A major problem with the *Enterococci* is that they are very resistant to antibiotics and have the ability to survive in harsh environments in the community and persist in hospital settings [3]. Because of this, they become important and responsible etiological agents in the community as well as healthcare-associated infections (HAIs), particularly in patients with prolonged hospital stays, severe underlying disease, or previous broad-spectrum antibiotic therapy [5, 6].

According to the World Health Organization (WHO) report in 2017, vancomycin-resistant Enterococci (VRE) is one of the most resistant bacteria in their “Global Priority list of antibiotic-resistant bacteria” [6, 7]. In the same manner, the Center for Disease Control and Prevention (CDC) has classified *Enterococci* among bacteria with a threat level of serious [8, 9].

During the last decade, a dramatic increase in the occurrence of vancomycin-resistant enterococci (VRE) has been noted in hospitals within the United Kingdom and the United States, and they have currently become the cause of one-third and one-fifth of all healthcare-associated infections in the United States and some European countries, respectively [10, 11].

Although the presence of VRE has been studied in many developed regions of the world, however there is a lack of comprehensive data indicating the burden of VRE in Africa, but few studies in South Africa showed 74.8% followed by Egypt 37.2%, Uganda 9.8%, Morocco 8.2%, and Ethiopia 7.9%, respectively [12].

*Enterococci* are the second bacteria to be reported from surgical wound infection and nosocomial urinary tract infection (UTI) and the third most frequently reported cause of bacteremia. Especially, *Enterococcus faecalis* and *Enterococcus faecium* have become causes of international concern [6].

At present, a serious public issue is the presence of multidrug-resistant bacteria including VRE and limited availability of drugs to treat VRE infections in the GIT as components of the normal microflora. The clinical significance of multidrug-resistant bacteria in the GIT is also documented by different researchers. In their study, an ESBL-positive strain of *Klebsiella pneumoniae* and *Enterobacter cloacae*, isolated from rectal swabs, were found to be identical with strains of VRE due to the transfer of the genetic determinant of vancomycin resistance to other Gram-positive and Gram-negative bacteria causing bacterial infections [8].

Asymptomatic VRE gut colonization precedes infection with susceptible hosts, such as patients who are exposed to multiple and prolonged courses of antimicrobial agents like human immunodeficiency virus (HIV)-infected individuals, severely ill, hospitalized for long lengths of stay, living in a long-term care facility, located in close proximity to another colonized or infected patient, or hospitalized in a room previously occupied by a patient colonized with VRE. Colonization is often obtained by vulnerable hosts in an environment with an increased rate of patient colonization with VRE [10, 13]. The colonization rate of VRE was reported in Europe, Asia, Australia, South America, and some African countries. However, there are no sufficient data available on the prevalence and risk factors of VRE in developing countries like Ethiopia. It also becomes a therapeutic challenge to physicians due to the ease of acquiring vancomycin-resistant genes and the presence of different selection pressures for VRE proliferation and rapid expansion of resistant populations [10]. Several studies have documented that *Enterococcal* infections are most commonly caused by the patient's own commensal flora [12]. Therefore, this study was conducted with the aim of determining the gastrointestinal colonization rate of vancomycin-resistant *Enterococci* among hospitalized patients at Hawassa University Comprehensive Specialized Hospital in Southern Ethiopia.

## 2. Methods

### 2.1. Study Design and Area

A prospective hospital-based cross-sectional study was conducted from April 1 to June 30, 2021, at Hawassa University Comprehensive Specialized Hospital (HUCSH) which is located in Hawassa city, Sidama Regional State, Ethiopia, located 275 kms from the capital city of Ethiopia Addis Ababa. According to projections of the central statistics authority of Ethiopia, Hawassa population is estimated to be 436,992 in 2012E.C [14]. Hawassa University Comprehensive Specialized Hospital is the only hospital in the region with more than 500 beds serving for about 18 million populations in the nearby regions of Oromiya, SNNPR, and Somalia. The hospital gives different outpatient and inpatient health care services such as HIV/AIDS care and treatment, oncology services, chronic disease management clinic, surgery, gynecology and obstetrics, internal medicine, pediatrics, ophthalmology, psychiatry, radiology, and pathology services.

### 2.2. Study Population

All patients hospitalized in the medical ward, surgical ward, pediatric ward, and adult intensive care unit for >48 hours were considered as a study population. Patients from whom consent and assent were obtained from parents/guardians were included in the study, whereas patients who were unable to provide specimens were excluded from the study. The sample size was estimated using a single population proportion formula assuming a prevalence of 5% vancomycin-resistant *Enterococci* reported from Jimma, Ethiopia [12], a confidence interval of 95%, and a 3% margin of error. After considering 10% for the nonresponse rate, the final sample size was 223.

To determine the proportion of sample size for five wards, the average number of hospitalized patients in the past three months (January to March 2021) before the study was considered. Accordingly, the total number of patients in 4 wards, medical ward, pediatric ward, surgical ward, and adult intensive care unit were 182, 66, 12, 94, and 10, respectively. The total sum of patient flow for the last three months was 2007. The sample size was proportionally allocated to each ward based on the number of hospitalized patients in each ward in the last three months.

### 2.3. Data Collection

An interviewer-administered structured questionnaire was used to document the patient's demographic and clinical details, which included age, sex, place of residence, occupation, educational level, marital status, medical history, clinical diagnosis, prior hospital/ICU admission, date of present admission to hospital and PICU, history of antibiotic usage, consistency of stool, hand washing habit, admission ward, length of hospital stay, the reason for admission, and use of the medical device.

### 2.4. Isolation and Identification of *Enterococci*

During the study period, about 223 patients who were admitted and stayed for >48 hrs and from whom 5 mg of fecal specimen was collected in sterile plastic containers were included in the study. The specimens were labeled with a unique number, date, and time of specimen collection and transferred to the microbiology laboratory of HUCSH within 30 minutes. In case of delay, the specimen was placed within Cary-Blair semisolid medium (Oxoid Ltd, Basingstoke, Hampshire, England). Finally, stool specimen was inoculated onto *Enterococcus* selective media, Bile Esculin azide agar containing 6 mg/L vancomycin (Hardy Diagnostics, Santa Maria), and incubated at 37°C. After 24 hours of incubation, the plates were observed for a colony of bacteria with black/brown colonies, were presumptively identified as *Enterococcus,* and confirmed to the species level based on Facklam and Collins standard biochemical tests [15].

Colonies of bacteria which were Gram-positive cocci and catalase-negative were further subcultured on Brain Heart Infusion broth containing 6.5% NaCl and incubated at 45°C for 24 hours; this was indicated by turbidity which confirms the identity of *Enterococci* [16]. Mannitol fermentation (mannitol salt agar containing 7.5% NaCl) and ampicillin susceptibility were used for the identification of *E. faecalis* from *E. faecium*. Accordingly, *E. faecalis* can grow on mannitol salt agar and ferment mannitol, whereas *E. faecium* are unable to ferment mannitol and resistant to ampicillin [13].

### 2.5. Methods of Detection of Vancomycin-Resistant *Enterococci*

Pure colonies of *Enterococci* which were isolated from culture were taken using a plastic swab and mixed in physiological saline until the turbidity matches 0.5 McFarland standards. By using a sterile cotton swab and dipping it into prepared suspension, we transferred and gently swabbed onto the surface of Mueller–Hinton agar (MHA); after 3 to 5 minutes, a 30 *μ*g vancomycin disc was placed on the surface of MHA and incubated aerobically at 37°C for 24 hours and inspected for a zone of inhibition. The diameter of zone of inhibition was measured using a ruler, a diameter of zone of inhibition zone ≤14 mm was considered resistant, 15-16 was considered intermediate, and a diameter of zone of inhibition ≥17 mm was considered susceptible.

The minimum inhibitory concentration (MIC) of vancomycin was determined by the agar dilution method for all the *Enterococcal* isolates grown on BEA as per the Clinical and Laboratory Standard Institute guidelines version 21 [17]. Similar McFarland standards' suspension was prepared to perform MIC, the suspension was prepared, vancomycin E-test (AB Biodisk, Solna, Sweden) was placed on the surface inoculated Mueller–Hinton agar and incubated aerobically at 37°C for 24 hours, and the MIC was read by observing the inhibition zone. The MIC values were interpreted based on the breakpoint recommended by CLSI. Values of MIC  ≤ 4 *μ*g/ml, 8–16 *μ*g/ml, and ≥32 *μ*g/ml were considered susceptible, intermediate, and resistant, respectively [17].

### 2.6. Antimicrobial Susceptibility Testing

The antimicrobial susceptibility of the isolates to other antibiotics, namely, penicillin (P) (10 IU), ampicillin (AMP) (10 *μ*g), (Gen) (10 *μ*g), erythromycin (ERY) (15 *μ*g), tetracycline (TE) (30 *μ*g), chloramphenicol (CL) (30 *μ*g), linezolid (30 *μ*g), and ciprofloxacin (CIP) (5 *μ*g), was also performed on Mueller–Hinton agar (MHA) (OXOID, UK) by the Kirby–Bauer disk diffusion technique as mentioned by the Clinical and Laboratory Standard Institute guideline 2021 [17].

### 2.7. Operational Definition

#### 2.7.1. *E. faecalis*

A strain of *Enterococcus* was susceptible to ampicillin and ferment mannitol on mannitol salt agar, while *E. faecium* strains were resistant to ampicillin and were unable to ferment mannitol [13].

#### 2.7.2. Multidrug-Resistance (MDR)

This is when a bacterium is nonsusceptible to at least one antimicrobial agent that belongs to three or more antimicrobial categories [18].

### 2.8. Data Quality Assurance

The questionnaire was pretested in a population representing 5% of the sample size at Adare General Hospital a week before the actual data collection. The quality of reagents and equipment was checked, and all the reagents were used according to manufacturer instructions. The data were collected by trained data collectors. Bacterial strains such as *S. aureus* ATCC 25923 and *E. faecalis* ATCC51299 were used to check the performance of culture media. During the preparation of a new batch of culture media, sterility was checked by incubating 5% of the batch at 35–37°C for 24 hours.

### 2.9. Data Processing and Analysis

Data entry and analysis were performed using SPSS version 25. Summary statistics were performed using frequencies and proportions for categorical data such as the sociodemographic and clinical characteristics of participants. Crude odds ratio (COR) and adjusted odds ratio (AOR) with a 95% confidence interval were computed using bivariable and multivariable binary logistic regression. Variables with a *p* < 0.25 in a bivariable analysis were selected for further analysis by multivariable binary logistic regression. Statistically, significant association was set at *p* < 0.05.

### 2.10. Ethical Considerations

This research was reviewed by the Institutional Review Board of Hawassa University College of Medicine and Health Science, and permission was obtained from the Institutional Review Board (IRB) of the College of Medicine and Health Sciences with (Reference number: IRB/149/13). Permission was granted by Hawassa University Comprehensive Specialized Hospital. Participation was voluntary based, confidentiality was ensured for each participant, and informed consent was secured before the start of each interview, and the stool sample was collected for each participant.

## 3. Results

### 3.1. Sociodemographic Characteristics of Participants

A total of 223 hospitalized inpatients were tested with 100 response rates, of which 123 (55.2%) were males and the median age of the study participants was 30 years with a range of 1–80 years. Most participants (40.8%) were in the age range of 15–30 years. More than 60% of study participants were urban dwellers (Table 1).

### 3.2. Clinical Characteristics of the Study Participants

The duration and history of previous hospital stay of the study participants were 213 (95.5%) who stayed for <2 weeks with an average stay in the hospital before sample collection being 6.5 days (with a standard deviation of ±3.84). The majority of cases (51.6%) of admission were recorded from the surgical ward. 10.8% were admitted to ICU. Among these, 76.7% of them underwent invasive procedure such as surgery or urinary catheterization (Table 2).

### 3.3. Vancomycin-Resistant *Enterococci* Colonization Rate

**Figure 1:**
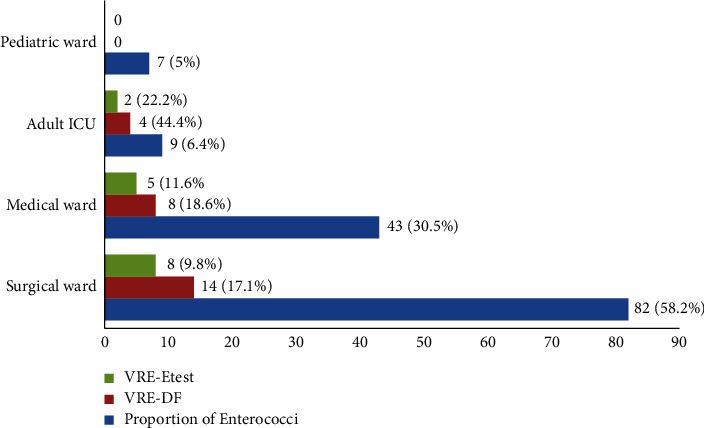
Distribution of *Enterococci* and VRE isolates in different wards and departments of HUCSH Sidama Regional State, Ethiopia. VRE = vancomycin resistant *Enterococci*.

From the total 223 stool specimens, 141 *Enterococci* species were isolated with a colonization rate of 63.2%. Among the isolated species, 65 (46.1%) and 76 (53.9%) were *E. faecalis* and *E. faecium*, respectively. The proportion of isolated species in different environments in the study area was higher in surgical ward followed by medical ward. Of the total 141 *Enterococci* isolates, 26 (18.4%) were vancomycin-resistant by the disk diffusion method. However, further check was performed for VRE by E-test strip tests; 15 out of 26 *Enterococci* were confirmed to be vancomycin-resistant by using E-test strips test. All of the 15 isolated VRE strains belong to *E. faecium*. The colonization rate of VRE using the *E*-test was 15 (6.7%) with 95% CI: (4.0, 10.6). A high proportion of VRE was detected in adults admitted to ICU (22.2%) followed by medical ward with a proportion of 11.6% (Figure 1).

The proportion of VRE among males whose age is > 50 years was 8.1% (Table 3). The proportion of VRE in the medical ward, surgical ward, pediatric ward, and adult intensive care units was 8 (5.7%), 14 (9.9%), 0, and 4 (2.8%), respectively, Based on *E*-test strips, the proportion of VRE in the abovementioned hospital wards was 5 (6.2%), 8 (7%), 0, and 2 (16.7%), respectively (Table 4).

### 3.4. Factors Associated with VRE Colonization Rate

Many variables were assessed for the presence or absence of association with VRE among hospitalized patients using both bivariate and multivariable logistic regression models. Accordingly, sociodemographic parameters such as sex, residence, and educational status and other clinical parameters such as length of hospitalization, ICU admission, history of treatment outside the hospital, and previous history of treatment with vancomycin were candidate variables for multivariable analysis.

In multivariable analysis, study participants who had no formal education (AOR = 4.26, 95% CI: 1.01, 18.06) were more likely to develop vancomycin-resistant *Enterococci* species (VRE) compared to those who had formal education. Based on their hospital stay, those who stayed for longer than two weeks (AOR = 4.10, 95% CI: 1.08, 15.57) were 4 times more likely to develop VRE as compared with their counterparts. Study participants who had a pervious history of treatment with vancomycin (AOR = 4.77, 95% CI: 1.26, 18.09) were also about 5 times more likely to develop VRE as compared with those who had no history of taking vancomycin and those who took any of the antibiotics (Table 5).

### 3.5. Antimicrobial Resistance Profile of *Enterococci*

Among 141 *Enterococci* isolated, 56.7%, 53.9%, 63.1%, and 70.2% were resistant to penicillin, ampicillin, erythromycin, and tetracycline, respectively, while 98.6% and 87.9% of *Enterococci* were susceptible to linezolid and chloramphenicol, respectively. The *E. faecalis* were resistant to erythromycin 69.7%, tetracycline 88.2%, and ciprofloxacin 69.7% (Table 6).

All VRE *E. faecium* were resistant to erythromycin; only one of them was susceptible to penicillin. However, 86.7% of them were susceptible to linezolid and chloramphenicol (Table 7).

### 3.6. Multidrug-Resistant Profile of *Enterococci*

Overall, the proportion of multidrug resistance (MDR) among *Enterococci* was 77 (54.6%). Most of the 23 (29.9%) were resistant to five antibiotics belonging to different classes, whereas 21 (27.3%) were resistant to four antibiotics, and 12 (15.6%) were resistant to six antibiotics that belong to different classes of antibiotics (Table 8).

## 4. Discussion

*Enterococci* are commensal of the gastrointestinal tract and are often multidrug-resistant; they may transfer antibiotic-resistant genes to other potentially pathogenic bacteria such as *S. aureus*. They themselves can cause disease in some circumstances, particularly in a hospital environment where patients with several underlying conditions reside. If VRE causes disease among colonized patients or other hospitalized patients it is difficult to manage as there are few treatment options [19, 20]. In the current study, 6.7% of hospitalized patients at HUCSH were colonized with VRE. A similar VRE colonization rate was reported from other parts of Ethiopia: Gondar (6.2%) [16], West Amhara (7.7%) [21], Addis Ababa (6.7%) [22], and Northwest Ethiopia (7.8%) [23]. The overall prevalence of *Enterococci* colonization in the current study was (63.2%) which was higher than the studies in Jimma, Ethiopia (23.08%) [24], and Dessie, Ethiopia (37.33%) [10]. However, this finding was lower than other studies, reported from Ethiopia (76−89%) [12, 24]. The majority of isolated species 76 (53.9%) were *E. faecium* followed by 65 (46.1%) *E*. *faecalis*. All vancomycin resistance belongs to *E. faecium* and emerging as the main nosocomial pathogen in the last two decades [25]. In some studies conducted in countries such as Turkey (1.55%) [26] and Nigeria (4.07%), the prevalence of VRE was lower than our finding [27]. This difference might be due to laboratory methods used (disk diffusion vs. MIC), variation in the study participants, effective use of infection prevention, antibiotic use, and source of specimen.

Long duration of hospital stay and use of antibiotics are among the frequently reported risk factors for VRE colonization and infection. The gradual increase and clonal expansion of VRE might have contributed to a higher prevalence [28, 29]. In addition to this, variation in the prevalence of VRE could be due to socioeconomic variation, exposure to antibiotics for a prolonged duration, and habit of antibiotic use.

Participants with no formal education were about 4 times more likely to be colonized with VRE than their counterparts. The finding was consistent with studies conducted in Nigeria [27], Ethiopia (925), and China [28]. Participants without formal education may have a tendency of using antibiotics without appropriate prescription and sharing of antibiotics, both of which could lead to the development of antibiotic resistance. Longer hospital stay for longer than two weeks during admission was another factor which was 4 times more likely to develop VRE. This result is in line with a study conducted among hospitalized patients in Jimma, Ethiopia [12].

Participants who had pervious history of treatment with vancomycin were also highly associated with developing VRE than their counterparts. Consistent finding was reported from Brazil [29] and Germany [30]. In another study conducted in Gondar, Ethiopia, reported that use of antibiotics also causes the emergence of VRE [16].

About 57% of *Enterococci* isolated in this study were resistant to penicillin which is higher than the resistance rate reported in India (22.8%) [31], Jimma (22.7%) [32], and Dessie (34.8%) [17]. 53.9% of *Enterococci* isolated showed resistance to ampicillin which is comparable with a report from Gaza 53.2% (535). The level of resistance to erythromycin in this study was (63.1%). This finding was higher than the study reported from Brazil 32.6% (). Also, a low proportion of resistance to tetracycline was reported from Brazil (17.3%) compared to the finding in this study which indicated 70.2% resistance [29]. The higher drug resistance profile in our study might be due to variations in sample size and study participants who were hospitalized patients exposed to different antibiotics.

Unlike our study (56.7%), higher penicillin-resistant *Enterococci* prevalence was reported from Gaza (71.3%), India (69.6%) [31], South India (89%) [33], Arbaminch (69.9%) [32], and Addis Ababa (80%) [12]. Reports from Iran (80%) [34] and Sothern India (86%) [33] showed high ampicillin-resistant *Enterococci* as compared to ours. In the current study, only 6.4% of *Enterococci* were resistant to chloramphenicol which was different from not in a report in India (96%) [31]. 70.2% of *Enterococci* isolated in this study were resistant to tetracycline which is high compared to reports from other parts of Ethiopia (34%) [19] and Iran (18%) [34], but higher resistance was reported from Gaza (80.9%). Moreover, most *E. faecium* isolated in the present study were resistant to erythromycin, tetracycline, and ciprofloxacin as compared to *E. faecalis*.

Almost all vancomycin-resistant *Enterococci* were resistant to ampicillin which showed a signal that they are acquired from a hospital environment. A similar study in the United States also indicated that VRE was reported only from health care settings only [35].

About 54.6% of *Enterococci* isolates were multidrug-resistant which is lower than the finding reported from Iraq (85.7%) [35]. In the opposite, lower MDR was reported from Dessie, Ethiopia (29.5%) [17]. The discrepancy of the findings might be due to variation in the geographical distribution of strain, trend, and frequency of antibiotic prescription, community antibiotic usage practice, and definition of MDR.

### 4.1. Limitation of the Study

The isolated *Enterococci* were not identified to the genetic level using molecular characterization due to resource limitation and budget constraints. Even though the study is conducted in a large hospital in the region and gives a service for different regions of Sidama SNNPR, Oromiya, and Somalia, it might be generalized to other hospital settings.

## 5. Conclusions

In our study, high incidence of vancomycin-resistant *Enterococci* was found. Previous exposure to antibiotics and hospital stay for more than two weeks were significant factors for vancomycin-resistant enterococci gut colonization. The study also showed that the isolated *Enterococci* had variable degrees of resistance to commonly prescribed antibiotics. Most *Enterococci* isolated were also showed resistant to one or more of the commonly prescribed antibiotics which leads to a common worldwide problem multidrug resistance. Therefore, periodic surveillance on antimicrobial resistance pattern, adhering to rational use of antibiotics and implementing infection prevention protocols may reduce colonization by VRE.

## Figures and Tables

**Table 1:** Sociodemographic characteristics of participant at Hawassa University Compressive Specialized Hospital, Sidama Regional State, Ethiopia (*N* = 223).

Variables	Category	Frequency (%)
Sex	Male	123 (55.2)
	Female	100 (44.8)

Age in years	<5	16 (7.2)
	5–14	10 (4.5)
	15–30	91 (40.8)
	31–50	77 (34.5)
	>50	29 (13)

Residence	Rural	83 (37.2)
	Urban	140 (62.8)

Educational status	No formal education	78 (35.0)
	Elementary	56 (25.1)
	High school	47 (21.1)
	Collage and above	42 (18.8)

Current occupation	Government employee	37 (16.6)
	Private employee	75 (33.6)
	House maker	59 (26.5)
	Student	41 (18.4)
	Day laborer	6 (2.7)
	Others	5 (2.2)

Marital status	Single	80 (35.9)
	Married	131 (58.7)
	Divorced	5 (2.2)
	Widowed	7 (3.1)

**Table 2:** Clinical characteristics of the study participants at HUCSH, Sidama Regional State, Ethiopia (*N* = 223).

Variables	Category	Frequency (%)
Consistency of stool	Formed	191 (85.7)
	Semiformed	26 (11.7)
	Diarrheic	6 (2.7)

Hand washing habit	≥3 times per day	150 (67.3)
	3 times per day	51 (22.9)
	No habit of washing	22 (9.9)

Admission ward	Medical ward	81 (36.3)
	Surgical ward	115 (51.6)
	Pediatric ward	15 (6.7)
	Adult intensive care units	12 (5.4)

Length of hospital stay	<2 weeks	191 (85.6)
	≥2 weeks	32 (14.4)

Admitted to ICU	Yes	32 (10.8)
	No	191 (85.6)

Underwent invasive procedure	Yes	171 (76.7)
	No	52 (23.3)

Reason for admission	Cancer	20 (9.0)
	Congestive heart failure	26 (11.7)
	Tuberculosis	19 (8.5)
	Blood clots	28 (12.6)
	Sever acute malnutrition	10 (4.5)
	Chronic disease	53 (23.8)
	Abdominal or cardiothoracic surgery	48 (21.5)
	Others	19 (8.5)

Use of medical device	Electronic thermometers	89 (39.9)
	Ear ox meters	5 (2.2)
	Stethoscopes	75 (33.6)
	Electronic thermometers and stethoscope	7 (3.1)
	Others	47 (21.1)

Patient relocated from one unit to other	Yes	101 (45.3)
	No	122 (54.70)

Treatment outside of the current hospital	Yes	96 (43)
	No	127 (57)

Had a history of vancomycin treatment	Yes	74 (33.2)
	No	149 (66.8)
	No	194 (87.0)

ICU: intensive care unit.

**Table 3:** The distribution of VRE within different sociodemographic parameters among hospitalized patients at HUCSH, Sidama Regional State, 2021 (*n* = 15).

Variables	Category	VRE	
		Yes *n* (%)	No *n* (%)
Age	<5	1 (6.25)	15 (93.75)
	5–14	0 (0)	10 (100)
	15–30	6 (6.59)	85 (93.41)
	31–50	5 (6.33)	72 (93.67)
	>50	3 (10.35)	26 (89.65)

Sex	Male	10 (8.1)	113 (91.9)
	Female	5 (5)	95 (95)

Age in years	<5	1 (6.3)	15 (93.7)
	5–14	—	10 (18)
	15–30	6 (6.6)	85 (93.4)
	31–50	5 (6.5)	72 (93.5)
	>50	3 (10.3)	26 (89.7)

Residence	Rural	9 (11.7)	68 (88.3)
	Urban	8 (5.7)	132 (94.3)

Educational status	No formal education	9 (13.9)	56 (86.2)
	Elementary and above	6 (3.8)	152 (96.2)

Current occupation	Government	2 (5.6)	34 (94.4)
	Private work	6 (10.7)	50 (89.3)
	House wife	2 (4.1)	47 (95.9)
	Student	2 (4.9)	39 (95.1)
	Day laborer	—	8 (18)
	Others	3 (9.1)	30 (90.9)

Marital status	Single	5 (6.3)	75 (93.7)
	Married	10 (7.6)	121 (92.4)
	Divorced	—	5 (18)
	Widowed	—	7 (18)

VRE: vancomycin-resistant *Enterococci*.

**Table 4:** Proportion of VRE among hospitalized patients with different clinical practices at HUCSH, Sidama Regional State, Ethiopia 2021 (*n* = 15).

Variables	Category	VRE	
		Yes *n* (%)	No *n* (%)
Consistency of stool	Formed	13 (6.8)	178 (93.2)
	Semiformed	1 (3.8)	25 (96.2)
	Diarrheic	1 (16.7)	5 (83.3)

Hand washing habit	≥3 times per day	8 (5.3)	142 (94.7)
	3 times per day	4 (7.8)	47 (92.2)
	No habit of washing hand	3 (13.6)	19 (86.4)

Admission ward	Medical ward	5 (6.2)	76 (93.8)
	Surgical ward	8 (20)	107 (93)
	Pediatric ward	—	15 (18)
	Adult intensive care units	2 (16.7)	10 (83.3)

Length of hospital stay	<2 weeks	8 (4.2)	183 (95.8)
	≥2 weeks	7 (21.9)	25 (78.1)

Admitted to ICU	Yes	5 (20.8)	19 (79.2)
	No	10 (5.0)	189 (95)

Underwent invasive procedure	Yes	13 (7.6)	158 (92.4)
	No	2 (3.8)	50 (96.2)

Reason/s for admission	Cancer	—	20 (18)
	Congestive heart failure	1 (3.8)	25 (96.2)
	Tuberculosis	2 (10.5)	17 (84.5)
	Blood clots	—	28 (18)
	Sever acute malnutrition	—	10 (18)
	Chronic disease	4 (7.5)	49 (92.5)
	Abdominal or cardiothoracic surgery	7 (14.6)	41 (85.4)
	Others	1 (5.3)	18 (994.7)

Use of medical equipment	Electronic thermometers	3 (3.4)	86 (96.6)
	Ear ox meters	—	5 (18)
	Stethoscopes	7 (9.3)	68 (92)
	Electronic thermometers and stethoscope	1 (14.3)	6 (85.7)
	Others	4 (8.5)	43 (91.5)

Patient relocated from one unit to other from	Yes	6 (5.9)	95 (94.1)
	No	9 (7.4)	113 (92.6)

Treatment outside of these hospital	Yes	9 (9.4)	87 (90.6)
	No	6 (4.7)	121 (5.3)

Had a history of treatment with vancomycin	Yes	6 (21)	143 (96)
	No	9 (12.2)	65 (87.8)

VRE: vancomycin-resistant *Enterococci*, ICU: intensive care unit.

**Table 5:** Bivariable and multivariate analysis of factors associated with VRE colonization rate among patients admitted HUCSH, Sidama Regional State, Ethiopia, 2021.

Variables	Category	VRE *n* (%)		COR (95% CI)	*p* value	AOR (95% CI)	*p* value
		Positive	Negative				
Sex	Male	10 (8.1)	113 (91.9)	1.681 (0.555, 5.090)	0.358		
	Female	5 (5.0)	95 (95.0)	1			

Residence	Rural	9 (11.7)	68 (88.3)	3.088 (1.056, 9.029)	0.039	2.139 (0.544, 8.415)	0.276
	Urban	6 (4.1)	140 (95.9)	1			

Educational status	No formal education	9 (13.8)	56 (86.2)	4.071 (1.386, 11.959)	0.011	4.258 (1.004, 18.057)	0.049
	Elementary and above	6 (3.8)	152 (96.2)	1		1	

Length of hospitalization	≤2 weeks	8 (4.2)	183 (95.8)	1		1	
	>2 weeks	7 (21.9)	25 (78.1)	6.405 (2.138, 19.186)	0.001	4.104 (1.082, 15.573)	0.038

Patient relocated from one unit to other	Yes	6 (5.9)	95 (94.1)	1			
	No	9 (7.4)	113 (92.6)	1.261 (0.433, 3.671)	0.670		

Admission in ICU	No	9 (4.7)	182 (95.3)	1		1	
	Yes	6 (18.8)	26 (81.3)	4.667 (1.535, 14.185)	0.007	3.908 (0.936, 16.320)	0.062

History of treatment outside this hospital	No	6 (4.7)	121 (95.3)	1		1	
	Yes	9 (9.4)	87 (90.6)	2.086 (0.716, 6.077)	0.178	1.882 (0.544, 6.508)	0.318

History of treatment with vancomycin	No	6 (4.0)	143 (96.0)	1		1	
	Yes	9 (12.2)	65 (87.8)	3.300 (1.128, 9.657)	0.029	4.765 (1.255, 18.093)	0.022

AOR: adjusted odd ratio, ICU: intensive care unit, CI: confidence interval, COR: crude odd ratio, VRE: vancomycin-resistant *enterococci*.

**Table 6:** Antimicrobial susceptibility patterns of *Enterococci* among patients admitted at HUCSH, Sidama, 2021 (*N* = 141).

Antimicrobial agents	Over all AST profile *n* (%)			AST profile of *E. faecalis n* (%)			AST profile of *E. faecium n* (%)		
	Susceptible	Intermediate	Resistant	Susceptible	Intermediate	Resistant	Susceptible	Intermediate	Resistant
Penicillin	61 (43.3)	—	80 (56.7)	56 (86.2)	—	9 (13.8)	5 (6.6)	—	71 (93.4)
Ampicillin	65 (46.1)	—	76 (53.9)	65 (18)	—	—	—	—	76 (18)
Erythromycin	17 (12.1)	35 (24.8)	89 (63.1)	15 (23.1)	25 (38.5)	25 (38.5)	2 (2.6)	10 (13.2)	64 (84.2)
Tetracycline	32 (22.7)	10 (7.1)	99 (70.2)	27 (41.5)	6 (9.2)	32 (49.3)	5 (6.6)	4 (5.2)	67 (88.2)
Chloramphenicol	124 (87.9)	8 (5.7)	9 (6.4)	62 (95.4)	1 (1.5)	2 (3.1)	62 (81.6)	7 (9.2)	7 (9.2)
Linezolid	139 (98.6)	—	2 (1.4)	65 (18)	—	—	74 (97.4)	—	2 (2.6)
Ciprofloxacin	37 (26.2)	45 (32)	59 (41.8)	29 (44.6)	30 (46.2)	6 (9.2)	8 (10.5)	15 (19.7)	53 (69.7)
Vancomycin	115 (81.6)	—	26 (18.4)	63 (96.9)	—	2 (3.1)	52 (69.4)	—	24 (31.6)

AST = antibiotic susceptibility testing.

**Table 7:** Antimicrobial resistance profile of VRE (*E. faecium*) recovered from hospitalized patients at Hawassa Comprehensive Specialized Hospital (*n* = 15).

Antimicrobial agents	VRE*-E. faecium* AST, *n* (%)		
	Susceptible	Intermediate	Resistant
Penicillin	1 (6.7)	—	14 (93.3)
Ampicillin	—	—	15 (18)
Erythromycin	—	—	15 (18)
Tetracycline	1 (6.7)	—	14 (93.3)
Chloramphenicol	8 (53.3)	4 (26.7)	3 (20)
Linezolid	13 (86.7)	—	2 (13.3)
Ciprofloxacin	1 (6.7)	—	14 (93.3)
Vancomycin	—	—	15 (18)

AST: antimicrobial susceptibility test, VRE: vancomycin-resistant *Enterococci*.

**Table 8:** Distribution of multidrug-resistant *Enterococcus* isolated from hospitalized patients at HUCSH, Sidama Regional State, Ethiopia, 2021 (*n* = 141).

Number of antibiotics	Combination of antibiotics	Isolates tested *n* (%)	Total MDR *n* (%)
R3	PEN, ERY, TET	1 (1.3)	8 (10.4)
	PEN, AMP, TET	2 (2.6)	
	PEN, AMP, ERY	1 (1.3)	
	PEN, AMP, CIP	3 (3.9)	
	AMP, TET, CIP	1 (1.3)	

R4	PEN, ERY, TET, CIP	5 (6.5)	21 (27.3)
	PEN, AMP, ERY, TET	12 (15.6)	
	AMP, VAN, ERY, E-T	1 (1.3)	
	PEN, AMP, TET, CIP	2 (2.6)	
	PEN, AMP, ERY, CIP	1 (1.3)	

R5	PEN, AMP, ERY, TET, CIP	21 (27.3)	23 (29.9)
	PEN, AMP, ERY, TET, CHL	1 (1.3)	
	VAN, ERY, TET, CHL, CIP	1 (1.3)	

R6	PEN, AMP, VAN, ERY, TET, CIP	8 (10.4)	12 (15.6)
	PEN, AMP, ERY, TET, CHL, CIP	2 (2.6)	
	PEN, AMP, VAN, ERY, TET, MIC	2 (2.6)	

R7	PEN, AMP, VAN, ERY, TET, MIC, CIP	9 (11.7)	10 (12.9)
	PAN, AMP, VAN, ERY, TET, CHL, CIP	1 (1.3)	

R8	PEN, AMP, VAN, ERY, TET, MIC, CIP	1 (1.3)	1 (1.3)

R9	PEN, AMP, VAN, ERY, TET, CHL, LIN, MIC, CIP	2 (2.6)	2 (2.6)

Total		77	77 (54.6%)

PEN: penicillin; AMP: ampicillin; VAN: vancomycin; ERY: erythromycin; TET: tetracycline; CHL: chloramphenicol, MIC: minimum inhibition concentration, LIN: linezolid, CIP: ciprofloxacin; R: resistance. MDR: organisms resistant to ≥3 antibiotics belonging to different classes.

## Data Availability

All data generated for this study were included in the manuscript and the data are available at the Hawassa University research and technology transfer directorate data base https://www.hu.edu.et/index.php/administration/vice-president-offices/research-and-technology-transfer.

## Abbreviations

VRE: Vancomycin-resistant *Enterococcus*
HUCSH: Hawassa University Comprehensive Specialized Hospital.
